# Supplementary material for: Inference of affordances and active motor control in simulated agents
Source: Front Neurorobot. 2022 Aug 11;16:881673. doi: 10.3389/fnbot.2022.881673 (PMC9405427; doi:10.3389/fnbot.2022.881673)
Supplement: Supplementary file 1 [file Presentation_1.pdf]

APPENDIX

1 MODEL AND TRAINING HYPERPARAMETERS AND DETAILS

Unless noted otherwise, we use the hyperparameters specified in this section. Visual input $\mathbf{v}$ consists of 11×11 pixels with the number of channels depending on the experiment. We obtain it by rasterization of a 0.5×0.5 square of the environment, centering and excluding the agent. Due to the maximum velocity of 0.23 units per time step, the agent's next position is always within it's visual field. Each channel corresponds to a property (obstacle, fog terrain, force field up and down) of the environment. The presence of a property is encoded with 1s, while the rest of the tensor is set to 0.

The vision model v_M is given by a CNN. It consists of a convolutional layer, a max pooling layer, and another convolutional layer followed by a fully connected layer. The convolutional layers have kernel size 3×3 with stride 1, no padding, 4 channels if $\dim(\mathbf{c}) < 32$, and 8 channels if $\dim(\mathbf{c}) = 32$. The max pooling layer has a receptive field size of 3×3 with stride 2. The fully connected layer has size 8 if $\dim(\mathbf{c}) < 5$, 16 if $5 < \dim(\mathbf{c}) \leq 16$, and 32 if $\dim(\mathbf{c}) = 32$. We use the $\tanh$ activation function in all layers. The vision model has

$$564 + \dim(\mathbf{i}) \cdot 36 + \dim(\mathbf{c}) \cdot 9, \text{ if } \dim(\mathbf{c}) < 5$$

$$964 + \dim(\mathbf{i}) \cdot 36 + \dim(\mathbf{c}) \cdot 17, \text{ if } 5 < \dim(\mathbf{c}) \leq 16$$

$$4312 + \dim(\mathbf{i}) \cdot 72, \text{ if } \dim(\mathbf{c}) = 32$$

parameters in total, where $\dim(\mathbf{i})$ denotes the number of channels of the input. We use Adam (Kingma and Ba, 2014) as our optimizer with learning rate 0.00075, β-values $(0.9, 0.999)$, and $\epsilon = 1e - 4$. In Experiment V, we use a 10th of the learning rate. We perform gradient clipping (Pascanu et al., 2013) and set the maximum norm to 2.

The transition model t_M is given by a MLP. The first fully connected layer has hidden size 32 with biases turned off. It is followed by the $\tanh$ activation function. The first of the two parallel fully connected layers predicts mean vectors with the linear activation function. A second parallel fully connected layer predicts vectors of standard deviations via the exponential activation function, providing non-negative values and therefore ensuring valid standard deviations. From a probabilistic point of view, under the assumption that the values before the activation function are uniformly distributed, these mappings implement an uninformative prior in a Bayesian framework (Nowlan and Hinton, 2018). The changes in position are scaled up by a constant factor of 4 before feeding them into t_M, such that it receives inputs which approximately cover the interval between -1 and 1. The transition model has

$$324 + \dim(\mathbf{c}) \cdot 32$$

parameters in total. We use Adam (Kingma and Ba, 2014) as our optimizer with learning rate 0.008, β-values $(0.9, 0.999)$, and $\epsilon = 1e - 4$. In Experiment V, we use a 10th of the learning rate. We perform gradient clipping (Pascanu et al., 2013) and set the maximum norm to 1.2.

We generate training data by sending randomly generated actions to the environment. Actions were generated in a way that ensures good coverage of the whole environment. For each environment used in our experiments, we gather 200 sequences of sensor-action-tuples. We use 160 sequences for training and 40 for validation. Each sequence has a length of 300 time steps. We train both components jointly

end-to-end with batch size 10 for 50 epochs in Experiments I-IV and for 500 epochs in Experiment V. We backpropagate the error through time every 50 time steps and reset the hidden states every 7 batches. This way we train the model to avoid exploding hidden states also during goal-directed control.

2 DETAILS ON PLANNING ALGORITHMS

When planning with gradient-based active inference, we apply the following adjustments to improve performance. Firstly, if an optimization cycle increases EFE, we perform early stopping and use the policy from the cycle before. Secondly, we decrease the learning rate exponentially over the policy from the future to the present. This leads to more stable paths since actions which lie in the later future are adapted more than actions to be executed in the nearer future. More precisely, given a mean learning rate α and decay γ, we set the learning rate for action $\mathbf{a}^{t+\tau}$ to:

$$\alpha_{\mathbf{a}^{t+\tau}} = \alpha \cdot \frac{\gamma^{P-\tau}}{\sum_{\tau}^{P} \gamma^{P-\tau}}$$

See Appendix 5 for a description of how to compute gradients when the objective is given by the FE between two multivariate normal distributions. After each update, we clamp the policy to be in the correct value range. Finally, after optimization, we shift the policy while copying the last element. We use stochastic gradient descent with learning rate 0.005, set the exponential learning rate decay to $\gamma = 0.9$, and perform 50 optimization cycles. If a policy update leads to worse performance, we stop the optimization and use the policy from before.

During evolutionary-based planning, we use normal distributions to model actions. In order to improve performance, we apply the following modifications. We use a momentum term on the means and covariances (De Boer et al., 2005). After a single optimization iteration, we keep a fixed number K of the elites for the next iteration (Pinneri et al., 2020). After optimization, we do not discard the means but shift them (Wang and Ba, 2019; Chua et al., 2018) while copying the last action in order to not start from scratch in the next optimization. We reset the variances, however, to avoid local minima. Analogously, we shift the elites that we keep (Pinneri et al., 2020). We use the first action from the best sampled policy as the optimization result (Pinneri et al., 2020). Instead of clipping sampled actions, we perform rejection sampling and sample until we have an action within the allowed value range. We generate 50 trajectory candidates, use 5 elites for parameters estimation, keep $K = 2$ elites for the next optimization cycle, use an initial covariance of 0.5, and a momentum of 0.1.

3 AFFORDANCE MAPS FROM EXPERIMENT III AFTER DIFFERENT AMOUNTS OF EPOCHS

Here, we show affordance maps from Experiment III (Subsection 4.5) after different amounts of epochs. In Figure 12 we see that with increasing amounts of training epochs, the upper and lower obstacles get encoded more similarly, the additional meaningless information gets more filtered out, and the affordance maps get more distinctive regarding the encoding of different behavioral possibilities.

4 DERIVATIVE OF NEGATIVE LOG-LIKELIHOOD IN A NORMAL DISTRIBUTION

In this section we derive the negative log-likelihood in a multivariate normal distribution with respect to the distribution's parameters.

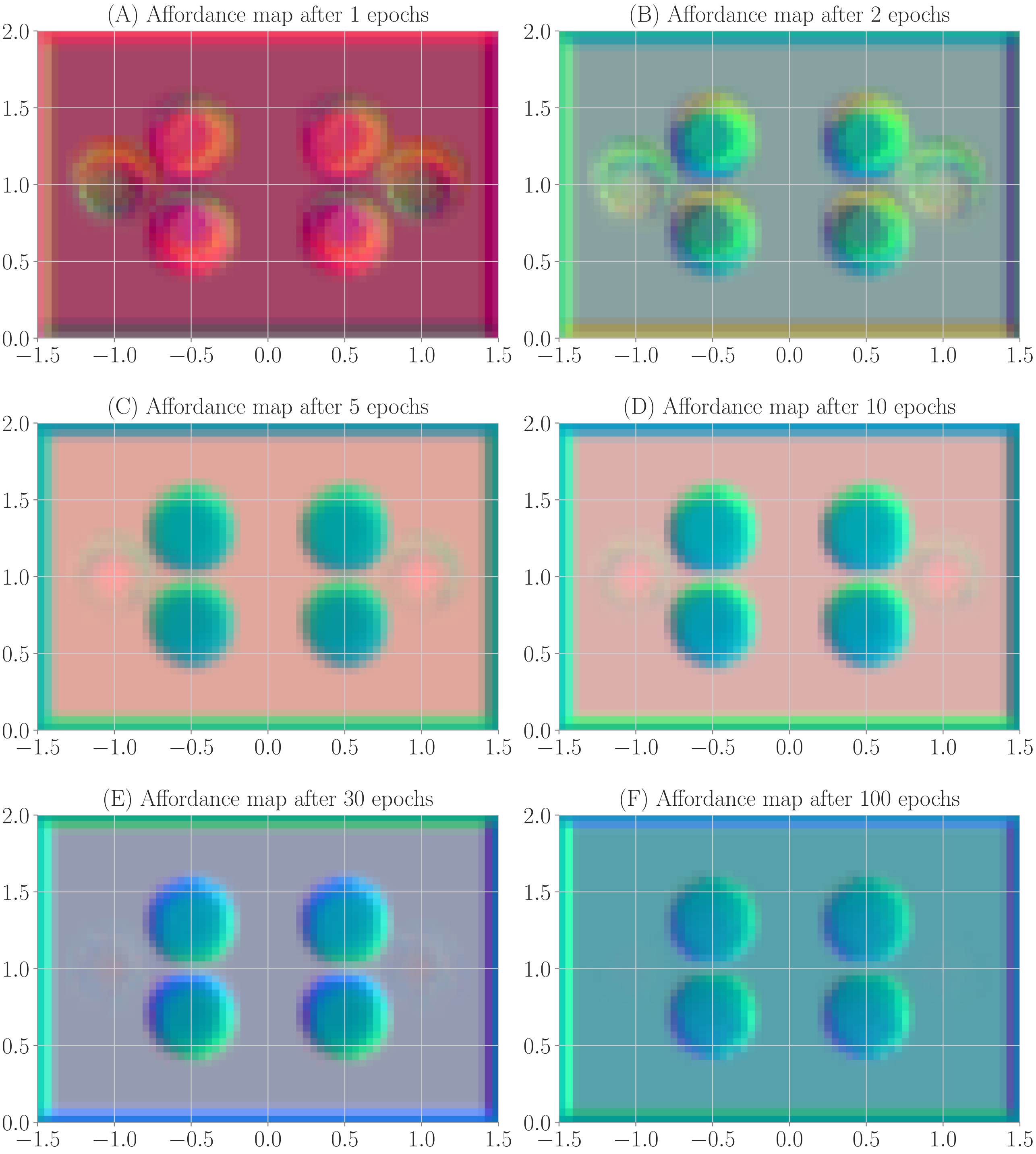

Figure 12. Exemplary affordance maps from Experiment III (Subsection 4.5) for context size 8 after different amounts of epochs. To generate these maps, we probed the environmental map at every sensible location, applied the vision model to each output, performed dimensionality reduction to 3 via PCA, and interpreted the results as RGB values.

695 The likelihood in a multivariate normal distribution is given by its probability density function:

$$\begin{aligned}\mathcal{L} &= p(\mathbf{x} \mid \mu, \mathbf{\Sigma}) \\ &= \mathcal{N}(\mathbf{x} \mid \mu, \mathbf{\Sigma}) \\ &= \frac{1}{\sqrt{(2\pi)^k |\mathbf{\Sigma}|}} \cdot e^{-\frac{1}{2}(\mathbf{x}-\mu)^T \mathbf{\Sigma}^{-1}(\mathbf{x}-\mu)}\end{aligned} \quad (10)$$

This leads to the following log-likelihood:

$$\begin{aligned}\mathcal{LL} &= \log 1 - \log\sqrt{(2\pi)^k|\boldsymbol{\Sigma}|} - \frac{1}{2}(\mathbf{x}-\mu)^T\boldsymbol{\Sigma}^{-1}(\mathbf{x}-\mu)\\ &= -\frac{1}{2}(k\cdot\log(2\pi)+\log|\boldsymbol{\Sigma}|+(\mathbf{x}-\mu)^T\boldsymbol{\Sigma}^{-1}(\mathbf{x}-\mu))\end{aligned} \tag{11}$$

Now, we take the derivative of the log-likelihood function with respect to the parameters of our probability distribution. The resulting quantity is also referred to as the *score*. We start by calculating the derivative with respect to the mean μ:

$$\begin{aligned}\frac{\partial\mathcal{LL}}{\partial\mu} &= \frac{\partial}{\partial\mu} - \frac{1}{2}(k\cdot\log(2\pi)+\log|\boldsymbol{\Sigma}|+(\mathbf{x}-\mu)^T\boldsymbol{\Sigma}^{-1}(\mathbf{x}-\mu))\\ &= -\frac{1}{2}(\frac{\partial(\mathbf{x}-\mu)^T\boldsymbol{\Sigma}^{-1}(\mathbf{x}-\mu)}{\partial\mu})\\ &= \boldsymbol{\Sigma}^{-1}(\mathbf{x}-\mu)\end{aligned} \tag{12}$$

Next, we calculate the derivative with respect to the covariance matrix $\boldsymbol{\Sigma}$. We assume $\boldsymbol{\Sigma}$ to be symmetric:

$$\begin{aligned}\frac{\partial\mathcal{LL}}{\partial\boldsymbol{\Sigma}} &= \frac{\partial}{\partial\boldsymbol{\Sigma}} - \frac{1}{2}(k\cdot\log(2\pi)+\log|\boldsymbol{\Sigma}|+(\mathbf{x}-\mu)^T\boldsymbol{\Sigma}^{-1}(\mathbf{x}-\mu))\\ &= -\frac{1}{2}(\frac{\partial\log|\boldsymbol{\Sigma}|}{\partial\boldsymbol{\Sigma}}+\frac{\partial(\mathbf{x}-\mu)^T\boldsymbol{\Sigma}^{-1}(\mathbf{x}-\mu)}{\partial\boldsymbol{\Sigma}})\\ &= -\frac{1}{2}(\boldsymbol{\Sigma}^{-1}-\boldsymbol{\Sigma}^{-1}(\mathbf{x}-\mu)(\mathbf{x}-\mu)^T\boldsymbol{\Sigma}^{-1})\end{aligned} \tag{13}$$

We are now able to calculate the derivatives of the log-likehood function of a multivariate normal distribution. In this work, we applied two simplifications: First, we used the special case of a bivariate normal distribution. Second, we assume all covariances to be 0, leading to a diagonal covariance matrix. With these assumptions, the multivariate normal distribution factors into two univariate normal distributions. We replace the covariance matrix $\boldsymbol{\Sigma}$ with a vector of variances σ^2 and end up with the following score:

$$\begin{aligned}\frac{\partial\mathcal{LL}}{\partial\mu_i} &= \frac{x_i-\mu_i}{\sigma_i^2}\\ \frac{\partial\mathcal{LL}}{\partial\sigma_i^2} &= \frac{1}{2\sigma_i^2}(\frac{1}{\sigma_i^2}(x_i-\mu_i)^2-1)\end{aligned} \tag{14}$$

5 DERIVATIVE OF FREE ENERGY BETWEEN NORMAL DISTRIBUTIONS

In this section we derive the expected free energy as used in this work between two multivariate normal distributions with respect to the parameters of one of the distributions. We first take the derivative of the entropy and subsequently of the divergence term.

The entropy of a multivariate normal distribution is given by:

$$\begin{aligned} H[p(\mathbf{x}|\mu, \mathbf{\Sigma})] &= \frac{1}{2}\log|2\pi e\mathbf{\Sigma}| \\ &= \frac{1}{2}(\log(2\pi e)^k + \log|\mathbf{\Sigma}|) \end{aligned} \tag{15}$$

Now we take the derivative with respect to the mean μ:

$$\begin{aligned} \frac{\partial}{\partial\mu}H[p(\mathbf{x} \mid \mu, \mathbf{\Sigma})] &= \frac{\partial}{\partial\mu}\frac{1}{2}(\log(2\pi e)^k + \log|\mathbf{\Sigma}|) \\ &= 0 \end{aligned} \tag{16}$$

Next, we take the derivative with respect to the covariance matrix $\mathbf{\Sigma}$ (assuming $\mathbf{\Sigma}$ to be symmetric):

$$\begin{aligned} \frac{\partial}{\partial\mathbf{\Sigma}}H[p(\mathbf{x} \mid \mu, \mathbf{\Sigma})] &= \frac{\partial}{\partial\mathbf{\Sigma}}\frac{1}{2}(\log(2\pi e)^k + \log|\mathbf{\Sigma}|) \\ &= \frac{1}{2}\frac{\partial\log|\mathbf{\Sigma}|}{\partial\mathbf{\Sigma}} \\ &= \frac{1}{2}\mathbf{\Sigma}^{-1} \end{aligned} \tag{17}$$

We are now able to calculate the derivative of the entropy of a multivariate normal distribution. Following the simplifications from above (Section 4), we again replace the covariance matrix $\mathbf{\Sigma}$ with a vector of variances σ^2 and end up with the following gradients:

$$\begin{aligned} \frac{\partial H[p(\mathbf{x} \mid \mu, \sigma)]}{\partial\mu_i} &= 0 \\ \frac{\partial H[p(\mathbf{x} \mid \mu, \sigma)]}{\partial\sigma_i^2} &= \frac{1}{2}\sigma_i^{-2} \end{aligned} \tag{18}$$

The Kullback-Leibler divergence between to multivariate normal distributions is given by:

$$\begin{aligned} D[p(\mathbf{x}_0 \mid \mu_0, \mathbf{\Sigma}_0) \mid\mid p(\mathbf{x}_1 \mid \mu_1, \mathbf{\Sigma}_1)] =& \frac{1}{2}(\mathrm{tr}(\mathbf{\Sigma}_1^{-1}\mathbf{\Sigma}_0) \\ &+ (\mu_1 - \mu_0)^T\mathbf{\Sigma}_1^{-1}(\mu_1 - \mu_0) \\ &- k - \log\frac{|\mathbf{\Sigma}_1|}{|\mathbf{\Sigma}_0|}) \end{aligned} \tag{19}$$

We first take the derivative with respect to the mean of the first distribution μ_0:

$$\begin{aligned} \frac{\partial}{\partial\mu_0}D[p(\mathbf{x}_0 \mid \mu_0, \mathbf{\Sigma}_0) \mid\mid p(\mathbf{x}_1 \mid \mu_1, \mathbf{\Sigma}_1)] &= \frac{\partial}{\partial\mu_0}\frac{1}{2}(\mu_1 - \mu_0)^T\mathbf{\Sigma}_1^{-1}(\mu_1 - \mu_0) \\ &= -\mathbf{\Sigma}_1^{-1}(\mu_1 - \mu_0) \end{aligned} \tag{20}$$

Now we take the derivative with respect to the covariance matrix of the first distribution $\mathbf{\Sigma}_0$ (assuming $\mathbf{\Sigma}_0$ and $\mathbf{\Sigma}_1$ to be symmetric):

$$\begin{aligned}\frac{\partial}{\partial \mathbf{\Sigma}_0} D[p(\mathbf{x}_0 \mid \mu_0, \mathbf{\Sigma}_0) \mid\mid p(\mathbf{x}_1 \mid \mu_1, \mathbf{\Sigma}_1)] &= \frac{\partial}{\partial \mathbf{\Sigma}_0} \frac{1}{2}(\mathrm{tr}(\mathbf{\Sigma}_1^{-1}\mathbf{\Sigma}_0) + \log \frac{|\mathbf{\Sigma}_1|}{|\mathbf{\Sigma}_0|}) \\ &= \frac{1}{2}(\mathbf{\Sigma}_1^{-1} - \mathbf{\Sigma}_0^{-1})\end{aligned} \tag{21}$$

We are now able to calculate the gradients of the Kullback-Leibler divergence between two multivariate normal distributions. Following the simplifications from above, we again replace the covariance matrices $\mathbf{\Sigma}_0$ and $\mathbf{\Sigma}_1$ with vectors of variances σ_0^2 and σ_1^2 and end up with the following gradients:

$$\begin{aligned}\frac{\partial D[p(\mathbf{x}_0 \mid \mu_0, \mathbf{\Sigma}_0) \mid\mid p(\mathbf{x}_1 \mid \mu_1, \mathbf{\Sigma}_1)]}{\partial \mu_{0,i}} &= -\sigma_{1,i}^{-2}(\mu_{1,i} - \mu_{0,i}) \\ \frac{\partial D[p(\mathbf{x}_0 \mid \mu_0, \mathbf{\Sigma}_0) \mid\mid p(\mathbf{x}_1 \mid \mu_1, \mathbf{\Sigma}_1)]}{\partial \sigma_{0,i}^2} &= \frac{1}{2}(\sigma_{1,i}^{-2} - \sigma_{0,i}^{-2})\end{aligned} \tag{22}$$

6 RELATIONSHIP BETWEEN NEGATIVE LOG-LIKELIHOOD AND KULLBACK-LEIBLER DIVERGENCE

In this work, we trained our architecture with the negative log-likelihood as the loss but performed goal-directed control via EFE minimization which includes the Kullback-Leibler divergence. Here, we show the relationship between the negative log-likelihood and the Kullback-Leibler divergence in general.

The Kullback-Leibler divergence between two probability distributions p and q is defined as

$$\begin{aligned}D[p(x) \mid\mid q(x)] &= E_{x \sim p(x)}\left[\log \frac{p(x)}{q(x)}\right] \\ &= E_{x \sim p(x)}[\log p(x) - \log q(x)] \\ &= E_{x \sim p(x)}[\log p(x)] - E_{x \sim p(x)}[\log q(x)]\end{aligned} \tag{23}$$

where E denotes the expected value. Let us now assume that P describes the distribution of some data we want to approximate with q. The left term does not depend on q and therefore is constant. If we now take N samples from the real distribution with $\lim_{N \to \infty}$ we end up with

$$-E_{x \sim p(x)}[\log q(x)] = -\frac{1}{N}\sum_{i}^{N} \log q(x) \tag{24}$$

which, up to a constant factor, is the definition of the negative log-likelihood.

We conclude that minimizing negative log-likelihood is equivalent to minimizing the Kullback-Leibler divergence.
